# A multivariable approach for risk markers from pooled molecular data with only partial overlap

**DOI:** 10.1186/s12881-019-0849-0

**Published:** 2019-07-19

**Authors:** Anne-Sophie Stelzer, Livia Maccioni, Aslihan Gerhold-Ay, Karin E. Smedby, Martin Schumacher, Alexandra Nieters, Harald Binder

**Affiliations:** 1 Forest Research Institute Baden-Württemberg (FVA), Wonnhaldestraße 4, Freiburg, 79100 Germany; 2 Institute for Medical Biometry and Statistics, Faculty of Medicine and Medical Center – University of Freiburg, Stefan-Meier-Straße 26, Freiburg, 79104 Germany; 3 Freiburg Center for Data Analysis and Modeling, University of Freiburg, Eckerstraße 1, Freiburg, 79104 Germany; 4 Center for Chronic Immunodeficiency, Faculty of Medicine and Medical Center – University of Freiburg, Breisacher Straße 115, Freiburg, 79106 Germany; 5 Institute of Medical Biostatistics, Epidemiology and Informatics, University Medical Center Johannes Gutenberg University Mainz, Obere Zahlbacher Straße 69, Mainz, 55131 Germany; 6 Department of Medicine, Solna (MedS), Eugeniahemmet, T2, Karolinska Universitetssjukhuset, Solna, Stockholm, 17176 Sweden

**Keywords:** Regularized regression, Single nucleotide polymorphism, Multivariable model, Partial overlap, Consortium

## Abstract

**Background:**

Increasingly, molecular measurements from multiple studies are pooled to identify risk scores, with only partial overlap of measurements available from different studies. Univariate analyses of such markers have routinely been performed in such settings using meta-analysis techniques in genome-wide association studies for identifying genetic risk scores. In contrast, multivariable techniques such as regularized regression, which might potentially be more powerful, are hampered by only partial overlap of available markers even when the pooling of individual level data is feasible for analysis. This cannot easily be addressed at a preprocessing level, as quality criteria in the different studies may result in differential availability of markers – even after imputation.

**Methods:**

Motivated by data from the InterLymph Consortium on risk factors for non-Hodgkin lymphoma, which exhibits these challenges, we adapted a regularized regression approach, componentwise boosting, for dealing with partial overlap in SNPs. This synthesis regression approach is combined with resampling to determine stable sets of single nucleotide polymorphisms, which could feed into a genetic risk score. The proposed approach is contrasted with univariate analyses, an application of the lasso, and with an analysis that discards studies causing the partial overlap. The question of statistical significance is faced with an approach called stability selection.

**Results:**

Using an excerpt of the data from the InterLymph Consortium on two specific subtypes of non-Hodgkin lymphoma, it is shown that componentwise boosting can take into account all applicable information from different SNPs, irrespective of whether they are covered by all investigated studies and for all individuals in the single studies. The results indicate increased power, even when studies that would be discarded in a complete case analysis only comprise a small proportion of individuals.

**Conclusions:**

Given the observed gains in power, the proposed approach can be recommended more generally whenever there is only partial overlap of molecular measurements obtained from pooled studies and/or missing data in single studies. A corresponding software implementation is available upon request.

**Trial registration:**

All involved studies have provided signed GWAS data submission certifications to the U.S. National Institute of Health and have been retrospectively registered.

**Electronic supplementary material:**

The online version of this article (10.1186/s12881-019-0849-0) contains supplementary material, which is available to authorized users.

## Background

An increasing number of high-dimensional molecular measurements from individuals are generated and data from such studies are frequently combined to identify markers of disease risk. For example, combining case-control studies with measurements of single nucleotide polymorphisms (SNPs) into large genome-wide association studies (GWAS) has allowed investigations into even very rare risk variants for some diseases [1]. Some of these consortia, such as the InterLymph Consortium on non-Hodgkin lymphoma (NHL) [2–9], not only allow for combining aggregate per-SNP statistics from each participating study, but provide individual level data from all studies for joint analysis. This opens the way for more sophisticated analyses, but any approach must contend with only partial overlap of the SNPs available from different studies due to differences in genotyping platform, quality control, and imputation approaches.

More and more multivariate methods for the analysis of high-dimensional case-control data arose in the past years. For example, [10] suggested an approach based on group lasso, and [11] considers a hybrid approach combining linear mixed models and sparse regression models, a so-called Bayesian sparse linear mixed model.

Further, regularized regression, such as the lasso [12] or componentwise boosting [13, 14], also provides an alternative to univariate approaches in that it takes SNP correlation structure into account and can directly provide genetic risk scores. [15] showed that those approaches outperform univariate analysis. Also, type 1 error control has recently been established for such approaches (see, e.g., [16, 17]), eliminating one of their major weaknesses. While univariate methods based on meta-analyses of per-SNP regression models can deal with partial overlap of SNP data in a straightforward manner, multivariable approaches typically require complete data on all individuals. This is often unfeasible in the context of large collaborative efforts.

Motivated by applications within the InterLymph Consortium, we addressed this issue by adapting a regularized regression approach, specifically componentwise boosting, for scenarios with partial overlap of SNP data and possibly differential missing individual level data per study. This is achieved by re-formulating the approach in terms of pairwise covariances, which can then be computed using all available SNP measurements. The focus of this article is to investigate how our methodology performs on a combined dataset from different studies, all enrolling their own individuals, and to contrast it with results from univariate analyses and an application of the lasso. See [18] on how to integrate multiple molecular sources in the presence of partial overlap in molecular data and individuals.

In the following, we briefly describe the data from the InterLymph Consortium and then propose the adaptation of componentwise boosting for synthesis regression in the Methods section. We also describe a stability selection approach for controlling the type 1 error. In the Results section, we illustrate the approach for the InterLymph data, in particular comparing its power to a naive approach that discards the studies causing the partial overlap as well as to univariate analyses. Finally, some discussion and concluding remarks on more general applicability in settings where data from several studies are to be combined, are provided.

## Methods

### The InterLymph application

The InterLymph Consortium (International Consortium of Investigators Working on Non-Hodgkin Lymphoma Epidemiologic Studies) is an open scientific forum for epidemiologic research on mature B-cell malignancies, including NHL. Formed in 2001, the Consortium is a group of international investigators who have completed or are in charge of ongoing case-control studies and who discuss and undertake collaborative research projects that pool data across studies to elucidate the etiology of lymphoma.

In the past few years, the genetics working group of the consortium has been engaged in large-scale GWAS, targeting among others the most prevalent NHL subtypes, chronic lymphocytic leukemia (CLL), diffuse large B-cell lymphoma (DLBCL), and follicular lymphoma (FL). For an investigation into the etiological relevance of genetic variability in epigenetic enzymes and regulators for NHL risk, the consortium provided imputed data for 366 pre-selected genes for all three subtypes from a total of 29 study sites, covering 8,628 cases and 8,748 controls. Part of this data restricted to the CLL and DLBCL subtypes will be used to illustrate the method developed here. Also, we pre-selected a specific chromosome, i.e. the results should not be interpreted from a biological perspective, but serve as illustration purposes of the proposed method. More comprehensive analyses from a subject matter perspective are ongoing.

In the InterLymph Consortium, the choice of different genotyping platforms, for example the Illumina OMNIexpress-24 BeadChip or the Illumina OMNI2.58 BeadChip, resulted in studies which lacked complete SNP overlap. In theory, imputing the data and performing an analysis based on the superset of all SNPs available in any of the studies would be favored. This can, however, not always be guaranteed because usually only high-quality imputed SNPs are taken into account. These may vary due to platform-specific differences in the coverage of genomic regions, which in turn leads to non-concordant SNPs.

### Synthesis regression

Molecular data from case-control designs are frequently analyzed by univariate approaches. Despite such initial univariate analyses, the markers identified from case-control studies frequently feed into multi-SNP genetic risk scores. Multivariable approaches that can perform variable selection are able to directly provide such risk scores, specifically taking correlation between markers into account.

The underlying idea in our setting is to construct a stable multivariable genetic risk score by selecting those SNPs that best explain the outcome. In such situations, regularized regression approaches can perform variable selection to obtain sparse models. Such approaches are widely used in high-dimensional data settings, when classical maximum likelihood estimation fails. Specifically for SNP data, approaches such as the lasso [12] or componentwise likelihood-based boosting [13] have been suggested. We use the latter as a basis for a synthesis regression approach [19] that can deal with partial overlap of the molecular data to address a challenge likely encountered when data are pooled from several studies, such as in the context of the InterLymph Consortium.

An advantage of componentwise boosting, compared to black-box approaches, is that it can be expressed in terms of univariate estimators. Therefore, we will briefly introduce the corresponding univariate estimators before subsequently describing componentwise boosting and its adaptation to partial overlap settings.

### The model and univariate estimators

In the following, we consider a set of in total *p* SNPs across *k* studies, the *superset* of all SNPs. Corresponding to a partial overlap scenario, let us further assume that covariate *j* (*j*=1,…,*p*) corresponding to a specific SNP is only present for *k*_*j*_ out of the *k* studies. Let *K*_*j*_={*l*∈{1,…,*k*}:covariate *j* is present for study *l*},|*K*_*j*_|=*k*_*j*_, be the set of studies comprising covariate *j*, and *n*_*l*_ the number of individuals in study *l*=1,…,*k*. Thus, in total, covariate *j* is present for $n_{j}=\sum \nolimits _{l\in K_{j}} n_{l}$ individuals.

We assume additive coding, e.g. SNP values are available as 0, 1, and 2. Therefore, we have a single covariate *x*_*lij*_ of a SNP *j*=1,…,*p* for patient *i*=1,…,*n*_*l*_ from study *l*=1,…,*k*. In the following, the SNP values are assumed to be centered and standardized, such that $\sum \nolimits _{i=1}^{n_{l}} x_{lij}^{2} = n_{l}$. Such a standardization to equal variance is not specific to the present proposal but is typical for regularized regression approaches.

Cases and controls are treated like in logistic regression to determine whether some markers occur more frequently in cases than in controls (and the other way around). In order to obtain such an outcome *y*_*li*_ for our regression model, the case-control status is coded as 1 for cases and −1 for controls and centered per study. The centering could be omitted, but it allows the intercept terms to subsequently be ignored. For simplified notation, we will still refer to values 1 and −1 in the following.

To investigate whether SNPs are linked to the case-control outcome, i.e. whether they should be considered as risk markers, we use a linear model

1 $$\begin{array}{*{20}l}  \mathbb{E}(Y=y|X=x)=x'\beta, \end{array} $$

where *x* is a vector comprising one or more of the SNP covariates, and *β* is a corresponding parameter that is to be estimated. This is non-standard, but allows for analytical tractability in the following. As we deal with a binary outcome, this is a quasi-likelihood approach, e.g. as compared to a logistic regression model. Yet, the linear model will typically provide non-zero estimates for *β* whenever they would also have been provided by a logistic regression model, i.e. the linear model should be sufficient for marker selection. At the same time, it enables a simple presentation and adaptation for partial overlap settings, as shown in the following.

If only a single SNP at a time is considered in model (1), a separate parameter $\hat {\beta }_{lj}$ is estimated for each SNP (*j*) and study (*l*), while the univariate estimate for *β*_*lj*_ takes the form

2 $$\begin{array}{*{20}l} \Delta_{lj}&=\frac{1}{n_{l}}\sum\limits_{i=1}^{n_{l}}x_{lij}y_{li} \end{array} $$

3 $$\begin{array}{*{20}l} &=\frac{1}{n_{l}}\sum\limits_{\substack{i\in\{1,\ldots,n_{l}\}:\\y_{i}=1}} x_{lij}- \frac{1}{n_{l}}\sum\limits_{\substack{i\in\{1,\ldots,{n_{l}}\}:\\y_{i}=-1}} x_{lij} \end{array} $$

being, up to a constant factor, the mean difference between SNP values in cases and SNP values in controls. This statistic can be pooled across studies, where a SNP is provided by using inverse variance weighting as has been established in a GWAS setting. The resulting joint statistic (up to a constant factor, assuming equal error variance) is

4 $$\begin{array}{*{20}l} \Delta_{j}&=\frac{1}{\sum\nolimits_{l \in K_{j}} {n_{l}}} \sum\limits_{l \in K_{j}} {n_{l}}\Delta_{lj} \end{array} $$

5 $$\begin{array}{*{20}l} &=\frac{1}{n_{j}} \sum\limits_{l \in K_{j}} \sum\limits_{i=1}^{n_{l}} x_{lij}y_{li}, \end{array} $$

i.e. an average of the per-study mean differences, corresponding to the calculation of the least squares estimates pooling all individuals where SNP *j* has been measured.

While such a statistic is not commonly used in practice, it is expected to result in SNP rankings similar to rankings obtained from standard statistics. The advantage of this non-standard statistic is that it provides a straightforward link to multivariable approaches, as shown in the following.

### Stagewise regression

Componentwise likelihood-based boosting [13] is a stagewise approach for estimating multivariable regression models, i.e. when *x* in model (1) comprises all SNPs. This approach performs variable selection by delivering estimates $\hat \beta =(\beta _{1},\ldots,\beta _{p})'$ with many elements equal to zero. It is closely linked to (forward) stagewise regression, being more cautious than classical (forward) stepwise selection, i.e. the final model is built in very small steps [20]. Due to this relation, the resulting variable selection is similar to the lasso, but tends to be more robust in the presence of strong linkage disequilibrium of the SNPs [13]. Therefore, we used this approach as a basis for synthesis regression in a setting with partial overlap.

The basic idea of componentwise likelihood-based boosting is to start with an initial estimate for the parameter vector *β* with all elements set to zero, i.e. none of the SNPs is part of the genetic risk score. Subsequently, in each of a number of steps, a single element of the parameter vector is selected to be updated when accounting for the SNPs that have been selected in earlier steps by an offset term, or equivalently, when considering the results from the previous step as an outcome. In doing so, the correlation between covariates is incorporated.

More formally, the boosting algorithm is as follows for each boosting step *m*=0,…,*M*:

1. For each covariate *j*, we determine the parameter estimate $\hat {\gamma }_{j}$ from a univariate regression model, taking previous boosting steps into account (more details given below).
2. Determine the index *j*^∗^ of covariate *j* with maximum value for $\left (\hat {\gamma }_{j}^{(m+1)}\right)^{2}$ which corresponds to the score statistic.
  To get a weak learner, set $\bar {\gamma }_{j}^{(m+1)}=\nu \cdot \hat {\gamma }_{j}^{(m+1)}$, where 0≤*ν*≤1 is a shrinkage parameter fixed in advance [21].
3. Update the parameter estimates
  6 $$ \hat{\beta}_{j}^{(m+1)}= \left\{\begin{array}{ll} \hat{\beta}_{j}^{(m)}+\bar{\gamma}_{j}^{(m+1)}&\text{ if } j=j^{*}\\ \hat{\beta}_{j}^{(m)}&\text{ else.}\\ \end{array}\right.  $$

This iterative procedure is stopped when the chosen stopping criterion is met. This could be, for example, a pre-defined number of covariates having non-zero estimates (the number of SNPs to be selected) or a pre-specified number of boosting steps [22].

We first consider the estimation per study, which requires specification of $\hat {\gamma }_{lj}^{(m+1)}$. A regression model for the residuals $r_{li}^{(m)}=y_{li} - \hat {y}_{li} = y_{li} - x_{li}'\beta ^{(m)}$ results in the following parameter estimate of the candidate model:

7 $$ \begin{aligned} \hat{\gamma}_{lj}^{(m+1)}=&\frac{1}{n_{l}} \sum\limits_{i=1}^{n_{l}} x_{lij}r_{li}^{(m)}\\ =&\frac{1}{n_{l}} \sum\limits_{i=1}^{n_{l}} x_{lij}\left(y_{li}-\hat{y}_{li}^{(m)}\right)\\ =&\frac{1}{n_{l}} \sum\limits_{i=1}^{n_{l}} x_{lij}y_{li}\\ &-\frac{1}{n_{l}}\sum\limits_{k:|\hat{\beta}_{k}^{(m)}|>0}\hat{\beta}_{k}^{(m)}\sum\limits_{i=1}^{n_{l}} x_{lij}x_{lik}\\ =&\Delta_{lj}-\frac{1}{n_{l}}\sum\limits_{k:|\hat{\beta}_{k}^{(m)}|>0}\hat{\beta}_{k}^{(m)}\sum\limits_{i=1}^{n_{l}} x_{lij}x_{lik}. \end{aligned}  $$

This can be interpreted as a decorrelation based on the estimated effects of the other SNPs, or alternatively as adjusting the (scaled) difference of means *Δ*_*lj*_ for effects that are due to other SNPs already included in the model.

Furthermore, this parameter estimate of the candidate model only depends on the univariate statistic *Δ*_*lj*_ and the (scaled) covariance $\frac {1}{n_{l}} \sum \nolimits _{i=1}^{n_{l}} x_{lij}x_{lik}$. This implies a straightforward way for estimating $\gamma _{j}^{(m+1)}$, pooled across studies where SNP *j* is available. Specifically, building on the univariate meta-analysis ideas described above, we propose using

8 $$ \begin{aligned} \hat{\gamma}_{j}^{(m+1)}&=\frac{1}{n_{j}} \sum\limits_{l \in K_{j}} \sum\limits_{i=1}^{n_{l}} x_{lij}y_{li}\\ &-\frac{1}{n_{j}}\sum\limits_{k:|\hat{\beta}_{k}^{(m)}|>0}\hat{\beta}_{k}^{(m)}\sum\limits_{l \in K_{j}} \sum\limits_{i=1}^{n_{l}} x_{lij}x_{lik}\\ &=\Delta_{j}-\frac{1}{n_{j}}\sum\limits_{k:|\hat{\beta}_{k}^{(m)}|>0}\hat{\beta}_{k}^{(m)}\sum\limits_{l \in K_{j}} \sum\limits_{i=1}^{n_{l}} x_{lij}x_{lik}, \end{aligned}  $$

i.e. not only the (scaled) differences are pooled, but also the covariances.

In this way, our proposal for synthesis regression is based only on pairwise covariances. This enables us to incorporate the data of several datasets at the same time. More precisely, all information on a specific covariate *j* that is available in the different studies can be utilized — irrespective of whether data for this covariate are available in only one, several, or all studies.

### Stability Selection

Application of covariance-based boosting for synthesis regression leads to a selection of SNPs from (pooled) molecular data. However, the approach itself does not allow for type 1 error control. The so-called *stability selection* [16] is a tool to approach the question of statistical significance in situations where subsampling is combined with variable selection. Judging the relevance of the (significant) effects is a different issue not considered in the scope of these investigations.

We refer to subsampling as a resampling method where *B* subsamples of all studies are drawn randomly without replacement[23]. The size of the subsamples is set to *n*/2, *n* being the size of the full sample. Below, we use the inclusion frequency (IF) to detail how frequently a SNP has been selected in all *B* subsamples.

The idea of the approach is to find out whether the variables selected more often than others over all subsamples are selected in a way that the type 1 error is controlled for. In the following, we will detail the approach, which can be directly applied to our synthesis regression proposal.

$\mathbb {E}(V)$, the expected number of false positives or *per-family error rate*, is bounded by a value determined from the resampled data and the variable selection procedure:

9 $$ \mathbb{E}(V)\leq\frac{1}{2\pi_{thr}-1}\cdot\frac{q^{2}}{p},  $$

where *V* is the number of false positives, *p* is the total number of covariates and *q* is the average number of selected covariates over all *B* subsamples in the last step *M* of the variable selection procedure [16]. *π*_*thr*_∈(0.5,1) denotes the threshold on the IF in *B* subsamples for calling a SNP significant. In general, different values for *π*_*thr*_ should be considered, as they correspond to different type 1 error levels.

When the chosen parameters and results from resampling provide for $\mathbb {E}(V)\leq 0.05$, the *familywise error rate*$\mathbb {P}(V\geq 1)$ is controlled at the 5% level since $\mathbb {P}(V\geq 1)\leq \mathbb {E}(V)\leq 0.05$.

## Results

In order to illustrate the use of covariance-based boosting as a synthesis regression approach in combination with stability selection, we use just an excerpt of the data from the InterLymph Consortium on CLL and DLBCL, two specific subtypes of NHL [3]. All analyses are based on SNP data for chromosome 9 still containing missing values for some SNPs even after imputation. The following section shows that by using the proposed method, all applicable information is taken into account during the analysis.

**Fig. 1:**
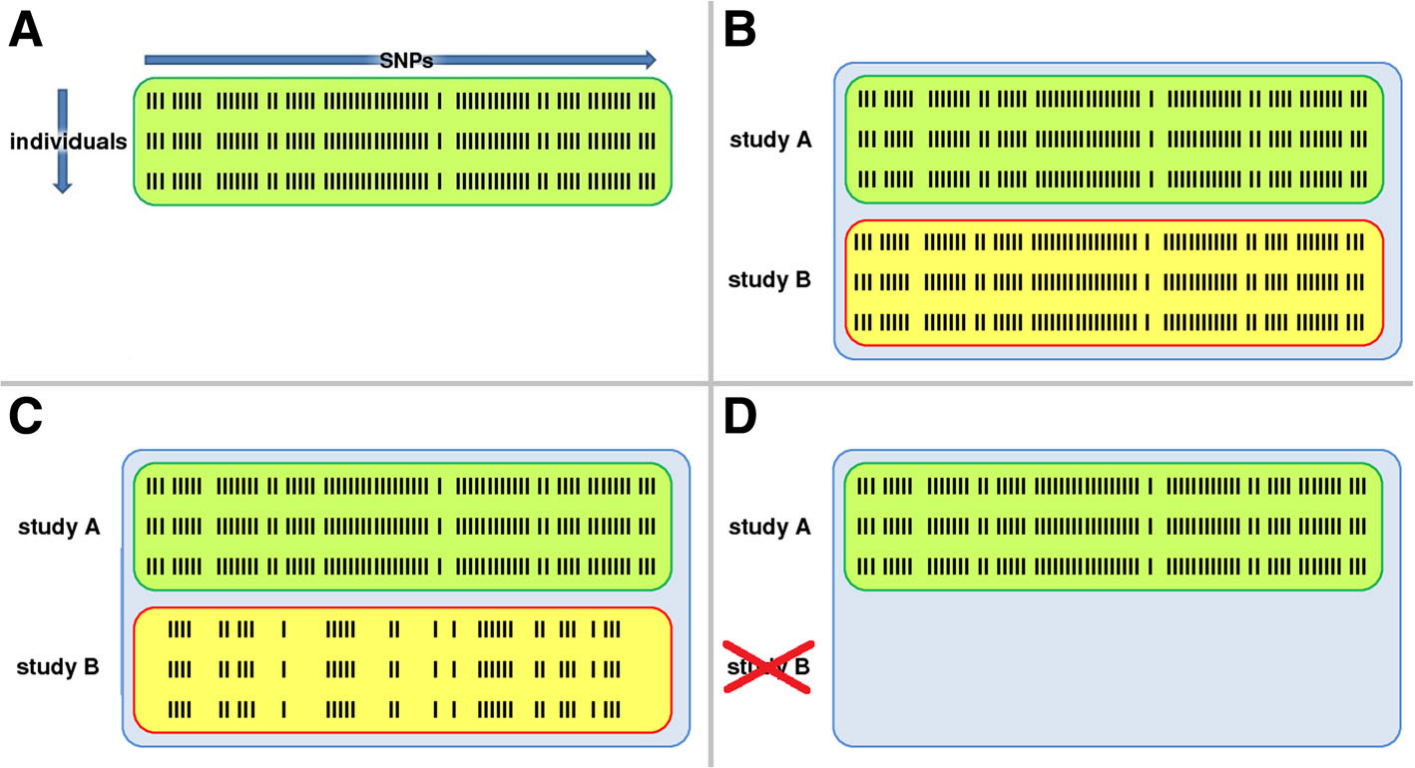
Scenarios appearing in the analysis of consortial data based on two studies after imputation. **a**. Illustration of SNP data for all individuals in a study. Every row contains all SNP data for one individual and each column represents the data for one SNP and all individuals. **b**. A perfect world: Both studies cover the same SNPs for all individuals (*full*). **c**. Reality: Differential coverage of SNPs in both studies. All SNPs in study B are a real subset of the SNPs in study A. An ideal analysis can use all applicable information (indicated by *red* for reduced). **d**. Reality: Differential coverage of SNPs in both studies as in Fig. 1c. In a complete case analysis, all information from study B is dropped (indicated by *part* for partial)

Figure 1 schematically shows different settings of SNP coverage for imputed SNP data when considering a combination of two studies, not showing potentially missing information for single SNPs per study. In Fig. 1b we consider a scenario where both studies comprise the same SNPs. Thus, even multivariable analysis approaches that require a complete case setting can be applied without problems if no missings are present. However, this is a "perfect world" setting.

The coverage of SNPs often differs between the studies due to different genotyping platforms. These differences often remain even after imputation. Depending on the multivariable analysis approach, an analysis might be able to incorporate all available information (Fig. 1c) or only provides a complete case analysis (Fig. 1d). For example, standard componentwise likelihood-based boosting would only use the complete case information as in Fig. 1d. Our newly developed boosting method can take into account all applicable information visualized in Fig. 1c, including information from individuals with missing values for single SNPs even after imputation due to inadequate imputation quality (not shown in Fig. 1 for convenience only). As stated previously, covariance-based boosting can also address other constellations where, e.g., no single study comprises all SNPs that are present in any of the investigated studies.

Subsequently, we will detail two specific applications of synthesis regression on data from the InterLymph Consortium to illustrate the consequences of different scenarios. The first considers artificial removal of some SNPs, where the analysis of the original data with synthesis regression is used as reference. To contrast synthesis regression with the lasso, we further applied both methods to the mode imputed data. The second application considers a combination of studies that truly have only partial overlap. In both scenarios, the number of boosting steps is set to 200 and we sample without replacement *n*/2 observations from the respective dataset (*n* observations).

### Application 1

Differential SNP coverage and considerably varying sample sizes are routine in consortial data. In a situation with two studies that differ extremely in sample size, study analysts may tend to ignore the small study and simply analyze the large study if the standard analysis approach can only be applied as complete case analysis. One aim of this application is to investigate the gains made by the possibility to analyze both, a *large study* (study A) and a *small study* (study B), with covariance-based boosting in comparison to analyzing only the *large study* (study A) with standard boosting. We further compare these analyses to the analysis in the scenario where both studies comprise the data for the superset of SNPs (“perfect world” scenario, see Fig. 1b), being referred to as *full* analysis hereafter, since the idea of our method is to recover the analysis of this full dataset. Therefore, we treat the SNPs identified by the *full* analysis as “truth”, regardless of their true biological meaning.

In order to illustrate the impact of these different settings on analysis results, we took the data from chromosome 9 of a DLBCL study in the InterLymph Consortium comprising 8,327 individuals and 15,961 SNPs according to genotyping platform 1 (GP1). We artificially separated this data into a *large study* (study A) comprising about 8/9 of the data (7,402 individuals) and a *small study* (study B) covering the other 1/9 of the data (925 individuals). In order to constitute differential SNP coverage, we further eliminated SNP information such that the *small study* (study B) data resembles SNP data from genotyping platform 2 (GP2), which is used in a small study of the InterLymph Consortium. For chromosome 9, GP2 covers 13,349 SNPs out of the 15,961 SNPs on GP1 (83.64 per cent).

For the *partial* analysis, we applied covariance-based boosting to the *large study* (study A) alone, that is 7,402 individuals with 15,961 SNPs, see Fig. 1d. In the *reduced* analysis we applied boosting to the *large study* as well as to the *small study* (study B), that is 7,402 individuals with 15,961 SNPs and 925 individuals with 13,349 SNPs, respectively. See Fig. 1c for an illustration. For the *full* analysis, we applied covariance-based boosting to the original data that is 8,327 individuals with 15,961 SNPs, see Fig. 1b. It is important to note that in the *full* analysis and in the *partial* analysis, covariance-based boosting does the same as standard componentwise likelihood-based boosting [21], because both scenarios contain complete case data.

Results for all three scenarios are shown in Table 1, where we took the 10 SNPs with the largest IFs according to the “truth” from the *full* data analysis, and also report their IFs from the *reduced* and *partial* data analysis, where we applied boosting with 200 steps on 100 subsamples, and *ν*=0.05 as shrinkage parameter. We further display the *p*-values from univariate analyses in the full data.

**Table 1:** Top 10 SNPs according to IFs for the *full* data analysis resembling the “truth” (IF_*full*_) in decreasing order

SNP		blackIF _*full*_	blackIF _*red*_	blackIF _*part*_	*p*-value
rs7039441	✓	0.68	**0.65**	**0.55**	0.05
rs1323398	✓	0.55	*0.63*	**0.49**	0.02
rs3793482	✘	0.44	**0.39**	**0.36**	0.02
rs1048251	✓	0.38	**0.27**	*0.40*	0.09
rs10965030	✘	0.28	**0.18**	**0.18**	0.07
rs10491695	✘	0.25	*0.50*	*0.50*	0.26
rs3750417	✘	0.22	**0.10**	**0.09**	0.06
rs7846927	✓	0.21	**0.12**	**0.19**	0.05
rs6477107	✓	0.19	*0.21*	*0.24*	0.02
rs12684584	✘	0.19	*0.21*	**0.18**	0.34

✓ SNP present in both studies

✘ SNP present in the *large study* (study A) but not in the white✘ *small study* (study B)

We additionally report the respective IFs from the *reduced* (IF_*red*_) and *partial* analysis (IF_*part*_). Numbers are marked in bold if the IF of the (*reduced* or *partial*) analysis is smaller than that of the *full* analysis (**IF<IF**_***full***_) and in italics if it is greater (IF>IF_*full*_). In 13 cases, we have **IF<IF**_***full***_, in 7 we have IF>IF_*full*_. We further report *p*-values from univariate logistic regression for each SNP

First of all, we see that the suggested procedure does work if we have partial overlap of SNP data between two studies: According to the results, 5 out of the 10 SNPs with the largest IFs in the *full* analysis are only present in the *large study* (study A). Accordingly, the other 5 SNPs are present in both studies.

Probably due to the correlation structures between the different SNPs, we find differences in IFs for the distinct SNPs over all three settings. However, we see that for most SNPs the results for the *reduced* analysis are closer or equally close to the results of the *full* analysis compared to those of the *partial* analysis.

In order to investigate the significance of our top hits, we additionally considered type 1 error control according to the stability selection framework. In our example, only the two top hits, rs7039441 and rs1323398, meet the requirement of IF >0.5 and thus are in principle candidates for stability selection. SNP rs7039441 has an IF of 0.68 in the *full* analysis and an IF of 0.65 in the *reduced* analysis. The total number of SNPs is *p*=15,961, an average number of selected SNPs in step 200 and all 100 subsamples *q*=16.93 in the *full* analysis, and *q*=16.69 in the *reduced* analysis. For illustration purposes we set the threshold for IFs *π*_*thr*_=0.65 and obtain

10 $$ \begin{aligned} \mathbb{E}(V)\leq&\ \frac{1}{2\pi_{thr}-1}\cdot\frac{q^{2}}{p_{super}}\\ =&\ \frac{1}{2\cdot0.65-1}\cdot\frac{16.93^{2}}{15,961}\\ =&\ 0.0599 \end{aligned}  $$

in the *full* analysis and $\mathbb {E}(V)\leq 0.0582$ in the *reduced* analysis, indicating that the expected number of false positives $\mathbb {E}(V)$ is not smaller than 0.05 in both cases (if the cutoff of 0.65 had been specified beforehand). However, it is close to 0.05 and thus indicates a potential for increased power compared to univariate testing, which does not account for multiple testing. SNP rs1323398 also does not meet the criterion for significance. Setting the threshold to 0.68 results in $\mathbb {E}(V)=0.0499$ for SNP rs7039441 in the *full* analysis.

To be able to contrast synthesis regression with the lasso, we applied both methods to a data set without any missings, as the lasso cannot deal with missing data – in contrast to synthesis regression. For the sake of simplicity, we used study A, the original dataset comprising all 8,349 individuals and 15,961 SNPs, and conducted mode imputation to replace all missing values (where about 25 per cent of the SNPs had a proportion of missing values of 50 per cent and more).

When applying synthesis regression to a dataset without any missings, our approach behaves just like standard componentwise boosting, as synthesis regression is simply a reformulation of the latter. In our application, a total of 831 SNPs were selected by boosting. We chose *λ*, the penalty coefficient in lasso, such that a total of 831 SNPs was also selected by the lasso. In total, 47 SNPs were selected by both analyses. We show those 6 SNPs that have been amongst the top 100 after application of both, the lasso and boosting, in Table 2. This is further contrasted with the inclusion frequency of these SNPs when applying synthesis regression to the original data from study A including missings, see IF _*full*_ in Table 1.

**Table 2:** Overlap of top 100 selected SNPs by the lasso and synthesis regression

SNP	rank _*lasso*_	rank _*Boosting*_	IF _*full*_
rs894243	12	5	0.14
rs80159021	21	1	0.00
rs7041984	25	9	0.00
rs7039441	32	40	0.68
rs7020755	60	4	0.00
rs6475560	71	30	0.00

The SNPs have been ordered in an increasing way according to their position in the selection sequence when applying the lasso with different values for *λ* (rank _*lasso*_). rank _*Boosting*_ details the SNP’s ranks according to the inclusion frequencies returned by the application of boosting. IF _*full*_ shows the inclusion frequencies when applying synthesis regression to the original study A data including missings

As indicated by the results, the lasso and boosting behave differently when being applied to the same data set without any missings. However, they still detect a considerable proportion of concordant SNPs compared to the large number of 15,961 SNPs that might potentially have been selected. The results gained by the application of synthesis regression to the original study A data shows again the top hit from the *full* analysis reported in Table 1. One further SNP is also identified by synthesis regression, while 4 SNPs receive inclusion frequencies equal to zero. Note that we used the same parameter setting for synthesis regression as for the *full* analysis, resulting in a selection of 290 SNPs in total.

### Application 2 based on data from two studies

In contrast to the application above, we now investigate how the method performs when applied to two different real studies at once. We took data from chromosome 9 for two CLL studies, study A with 8,349 individuals and study B with 726 individuals. These studies have a partial overlap in SNPs since different genotyping platforms (GP1 for the former and GP2 for the latter) were applied, resulting in 15,961 SNPs in study A and a subset of them comprising 13,349 SNPs in study B. This setting corresponds to the scenario depicted in Fig. 1c.

We performed a *combined* analysis using data from both studies. As a comparison, we also applied covariance-based boosting to both studies separately. In all settings, we applied boosting with 200 steps on 100 subsamples, and *ν*=0.1 as shrinkage parameter. Results for all three analyses are shown in Table 3, where we report the 10 SNPs with the largest IFs for the *combined* analysis and also state IFs for the respective SNPs in studies A and B, and *p*-values from univariate analyses in study A. Notably, covariance-based boosting is required for the *combined* analysis, while the analyses of both studies separately could also be performed with standard boosting.

**Table 3:** Top 10 SNPs according to IFs for the *combined* data analysis (IF_*comb*_) in decreasing order

SNP		IF _*comb*_	IF _*A*_	IF _*B*_	*p*-value
rs2274095	✘	0.52	**0.51**	-	0.42
rs722628	✓	0.48	**0.22**	**0.21**	0.55
rs7022345	✓	0.44	**0.40**	**0.07**	0.02
rs1323398	✓	0.41	**0.37**	**0.10**	0.13
rs2792232	✓	0.39	**0.32**	**0.10**	0.20
rs1886261	✘	0.35	**0.29**	-	0.20
rs10974947	✓	0.34	*0.42*	**0.13**	0.06
rs4742308	✓	0.34	**0.15**	**0.17**	0.31
rs4742247	✓	0.30	**0.14**	**0.06**	0.90
rs7018851	✓	0.29	**0.19**	*0.37*	0.63

✓ SNP present in both studies

✘ SNP present in study A but not in study B

We additionally report the respective IFs from the analysis of study *A* (IF_*A*_) and study *B* (IF_*B*_). Numbers are marked in bold if the IF of the analysis (of study A or study B) is smaller than that of the *combined* analysis (**IF<IF**_***comb***_) and in italics if it is greater (IF>IF_*comb*_). For most SNPs, we have **IF<IF**_***comb***_, for only few we have IF>IF_*comb*_. We further report *p*-values from univariate logistic regression for each SNP

Similar as in Application 1, our proposed method succeeds in still detecting some SNPs that are only present in one study, study A, when performing the *combined* analysis. For these SNPs (rs2274095 and rs1886261) the missing information in study B does not lead to a substantial reduction of IF in the *combined* analysis compared to those in the analysis of study A alone. For less frequently selected SNPs of the *combined* analysis, we also found constellations where in study A alone the IF is equal to or higher than the IF in the combined analysis when considering SNPs that are not present in study B (results not shown).

There are quite many situations where the IF in the *combined* analysis exceeds those in both separate analyses (rs722628, rs7022345, rs1323398, rs2792232, rs4742308 and rs4742247). This might result from a gain in information across both studies involved and related correlation structures. For rs7018851 we see that the IF in the *combined* analysis is lower than in the analysis of study B alone. This is probably due to the differences in sample sizes between both studies, where the information from study A overlays that from study B. With rs10974947 we detect a SNP that is rarely selected in study B, but is selected very often in study A alone. This results in a lower IF in the *combined* analysis compared to the analysis of study A.

In the combined analysis and the analysis of study A alone, SNP rs2274095 reaches an IF>0.5. In the analysis of study B, two SNPs, rs6477134 and rs10815532, reach an IF>0.5. For all four inclusion frequencies we get $\mathbb {E}(V)>0.05$, indicating that these SNPs are not significant according to stability selection [16]. In the univariate analysis of study B we find an association of rs10815532 with case-control status which does not survive Bonferroni correction. In the univariate analysis of SNP rs2274095 in study A and rs6477134 in study B, even the unadjusted *p*-values are >0.05.

No other SNP reaches an IF >0.5 in any of the analyses, so we could not apply stability selection to them. Also, none of the univariate *p*-values remains significant after Bonferroni correction for multiple testing in the analysis of study A or study B.

To preclude that the sample size is the main driver for the selection of SNPs, we ran another analysis based on studies A and B, taking a random subset of 363 samples from the large study A, being half the sample size of the small study B (*n*=726). SNPs only present in study A and having a high IF in the analysis using the complete study A data still had high IFs when only using the randomly selected small subset of individuals from study A.

**Fig. 2:**
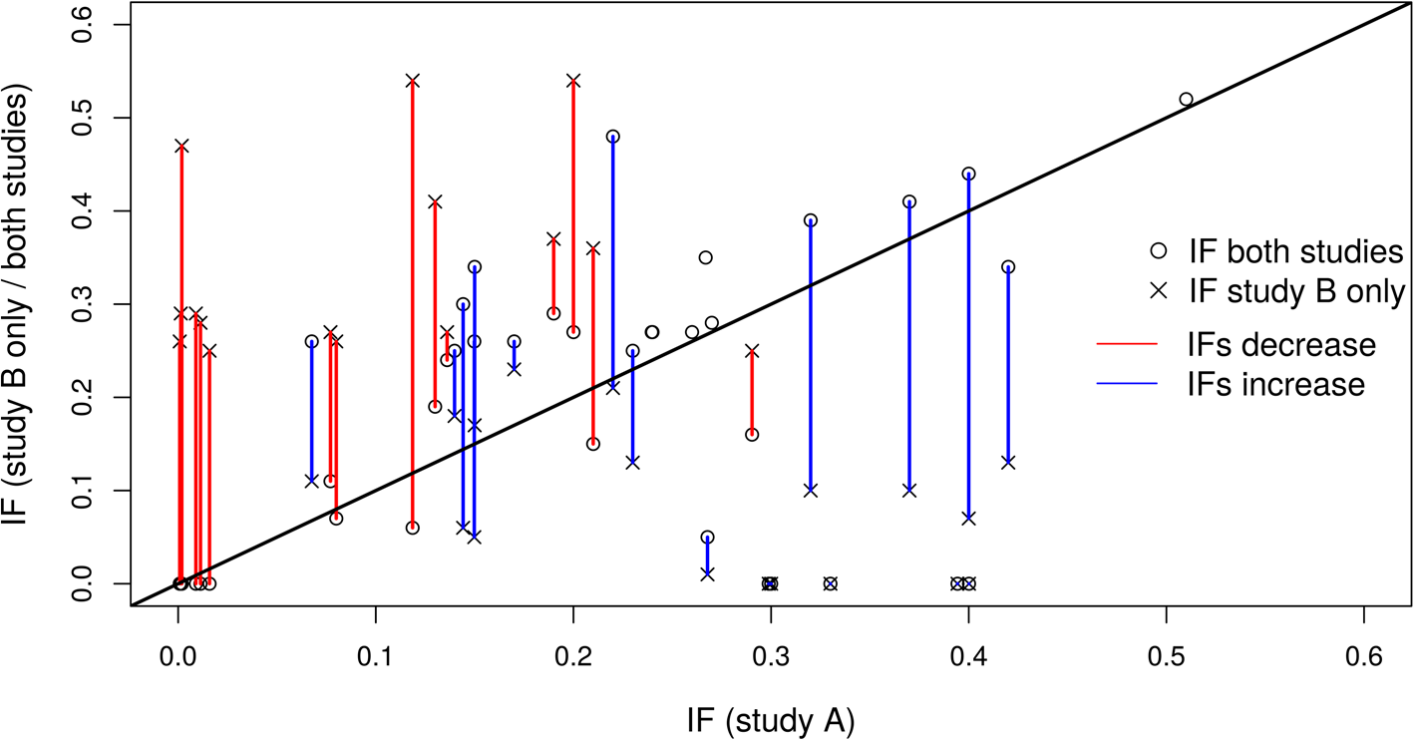
This illustration shows how combining information from both studies A and B changes the inclusion frequency (IF) in comparison to IFs in both single studies

Figure 2 illustrates how IFs decrease or increase when information from both studies are combined in comparison to IFs in the single studies for all SNPs having an IF ≥0.25 in any of the three analyses. The blue vertical lines indicate that IFs in the *combined* analysis are larger than IFs in the analysis of study B alone, while a reduction in IFs is indicated by a red vertical line. Therefore, a blue vertical line crossing the diagonal indicates that the IF in the *combined* analysis is higher than the IF in the analysis of study A, while a red vertical line crossing the diagonal indicates that the IF in the analysis of study A is higher than the IF in the *combined* analysis. For some SNPs, there is a notable decrease in IFs for the *combined* analysis compared to the analysis of study B alone. This decrease seems to occur mostly for those SNPs that have a rather small IF in study A. On the other hand, there is an increase in IFs for SNPs having a rather low IF in study B but a quite high IF in study A. For some SNPs with a higher IF in study A, the IFs are zero in both, the analysis of study B only and of both studies. In these cases, the missing signal in the smaller study B seems to superpose the signal from the larger study A.

### Computation time

For both applications we ran the code in parallel on 22 cores of 2x Xeon E5-2690v4, a 64 bit server providing 2.6 GHz and 512 GB memory.

In Application 1, each of the three analyses was conducted in 278.62 seconds on average. Runtime was 301.24 seconds for the *full* analysis, 274.74 seconds for the *reduced* analysis and 259.89 seconds for the *partial* analysis.

For Application 2, runtime was 206.93 seconds on average while it took 287.31 seconds for the analysis of study A, only 26.46 seconds for study B and 307.01 seconds for the combined analysis.

These results indicate that computation time roughly increases linearly with the number of individuals (when assuming similar numbers of SNPs) for the distinct analyses.

## Discussion

Regularized regression techniques with automated variable selection entail the promise of (i) potentially increasing power by taking correlation into account and of (ii) directly developing genetic risk scores from original individual level SNP data in consortia of several studies. Unfortunately, in practice this is hindered by only partial overlap of SNPs between studies, as exemplarily illustrated in an application based on an NHL dataset.

While there has been a recent surge in methods that perform integrative analysis of several datasets, none of these approaches addresses the problem present in our application. Such integrative approaches allow, for example, for the integration of multiple molecular sources into a clinical risk prediction signature [18] or the use of integrative regression networks for genomic association studies [24]. Yet, as stated, these methods do not allow for combining data with partial overlap. The closest candidate is a specific synthesis regression approach [19], which is only applicable in low-dimensional settings. In contrast, the current proposal is a synthesis regression approach that can deal with partial overlap in high-dimensional data. An additional asset is that it can also cope with missing data, i.e. all available information can be taken into account. This shows the great potential of the presented method as there is no “gold standard” for variable selection in high-dimensional data with missings so far. Being forced to use a complete case analysis in high-dimensional data with missings quickly becomes problematic: very few or even no observations might be left after removal of those individuals with at least one missing information. Besides the theoretical considerations, our applications could also show that SNPs not being present in all studies are selected by synthesis regression in practice.

The ability of synthesis regression to deal with missing data was accomplished by adapting a specific regularized regression approach, i.e. componentwise boosting. Specifically, the estimation in this approach could be expressed in terms of pairwise SNP covariances, which can be computed based on those studies for which a respective pair of SNPs is available. This method provides equivalent solutions in situations with complete SNP overlap and comes at no additional computational cost. For data without missings, the lasso is an alternative way to perform variable selection. We contrasted synthesis regression with the lasso in one application with complete data. However, since an extensive methods comparison between componentwise boosting and the lasso is not within the scope of this manuscript, we refer to [20, 25] in this regard.

Applied to genetic data on NHL case-control studies, the adapted boosting technique was combined with a resampling approach to stably identify SNPs for a genetic risk prediction signature. The corresponding resampling inclusion frequencies for each SNP indicated that considerable gains in stability can be obtained compared to just restricting the analysis to complete data. This can be explained by the additional data and related correlation structures across all involved studies. In some situations with extremely varying sample sizes, information from the large studies may overlay information from the small studies. But, depending on the correlation structures, even information from the small studies might contribute to a higher inclusion frequency in the combined analysis as shown in Application 2.

## Conclusions

In summary, our proposal removes a grave obstacle for using regularized regression techniques in large consortia, and thus opens the way for taking the correlation structure of SNPs into account right from the selection stage. Therefore, this innovative method potentially contributes to the development of improved genetic risk scores and should also be considered for other applications where molecular data from several studies are to be combined.

## Additional file

Additional file 1 A list of ethics committees which approved the studies providing data for our applications. (XLSX 11 kb)

## Data Availability

A platform-independent software implementation is available upon request. The original data that underlie the findings of this study are available from the InterLymph Consortium.

## Abbreviations

CLL: Chronic lymphocytic leukemia
DLBCL: Diffuse large B-cell lymphoma
FL: Follicular lymphoma
GWAS: Genome-wide association study
IF: Inclusion frequency
InterLymph Consortium: International consortium of investigators working on non-hodgkin lymphoma epidemiologic studies
NHL: Non-hodgkin lymphoma
SNP: Single nucleotide polymorphism
